# Bacterial load in meconium

**DOI:** 10.1002/imt2.173

**Published:** 2024-02-13

**Authors:** Wen‐Yu Jin, Jing Peng, Jinping Dai, Rongkang Tang, Jia‐Xin Guo, Huan Zhao, Jielin Wang, Shu Zhang, Yi‐Zhou Gao

**Affiliations:** ^1^ The Center for Microbes, Development and Health, Shanghai Institute of Immunity and Infection Chinese Academy of Sciences Shanghai China; ^2^ University of Chinese Academy of Sciences Beijing China; ^3^ Obstetrics and Gynecology Hospital of Fudan University Shanghai China; ^4^ Department of Oncology The First Affiliated Hospital of Zhengzhou University Zhengzhou China; ^5^ Hongqiao International Institute of Medicine, Tongren Hospital Shanghai Jiao Tong University School of Medicine Shanghai China; ^6^ Department of Gynecological Oncology Fudan University Shanghai Cancer Center Shanghai China

## Abstract

The spike‐in plasmid method was utilized to perform an analysis on meconium and second‐pass feces, yielding both relative and absolute quantitative results. With the absolute quantitative data, the abundance of bacteria in 17 meconium samples and 17 second‐pass fecal samples were found to be 1.14 × 10^7^ and 1.59 × 10^9^ copies/g, respectively. The mode of delivery can significantly influence the alterations and compositions of gut bacteria in a newborn within 72 h.

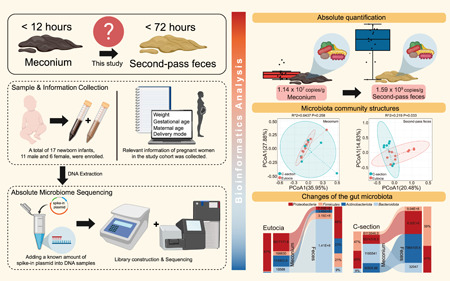

To the Editor,

The intestinal microbiota is host to an exceedingly large number of bacteria, estimated to be about 10^11^ CFU/g in the adult colon [1]. With the development of metagenomics, metabolomics, and other omics technologies [2], the composition and function of gut microbes have been further analyzed and recognized [3]. Existing studies have shown that metabolites of the gut microbiota may be associated with the development of a variety of diseases [4]. Moreover, the gut microbiome during infancy plays a pivotal role in human growth and development. Researchers speculate that a significant association exists between the infantile intestinal microbiota and the maturation of the infantile immune system, including the development of immune‐related disorders [5].

It is widely accepted that the composition of gut microbes undergoes the most significant changes during infancy, particularly between the ages of 1 and 3 months [6], stabilizing within the first year of life and maturing by approximately 5 years of age [7]. In previous studies, prenatal feces have been shown to be totally sterile [8]. Shifting the sight to the meconium, which determined as the first stool passed by the newborn and the color was totally black. After the first feeding, food stays in the infant's stomach for 2–3 h and in the small intestine for 8–96 h [9], and the meconium would gradually turn green. After 4 days of feeding, the feces would become yellow. Bacteria have been shown to be present in the meconium after the baby was born. However, the changes in the microbiome of meconium collected within 12 h post‐birth following the initial feeding stage remain unknown.

The composition of microorganisms in meconium has been studied in previous research using 16S ribosomal DNA (rDNA) sequencing [10]. However, traditional 16S rDNA sequencing or metagenomics is typically employed for samples containing a high abundance of bacteria. Given the exceptionally low microbial count in meconium, the background noise inherent in conventional sequencing methods could substantially influence the outcomes. Furthermore, contamination may occur during sample collection and instrumental analysis, making it difficult to distinguish the background signal from the real signal during analysis.

In recent years, there has been a growing interest in absolute quantitative omics analysis methods [11]. In this study, we aimed to investigate the presence of microorganisms in meconium and their dynamics within 72 h after birth. we conducted a screening of pregnant women who were healthy during their prenatal period, had no enteric‐related diseases, and had not been exposed to short‐term antibiotics. We collected fetal meconium excreted within 12 h of birth as well as the second‐pass feces within 72 h. Subsequently, we separately sequenced the 16S rDNA of each sample and analyzed them using internal standard plasmids. Additionally, we examined the differences in the fecal microbial composition of infants born via C‐section and eutocia. The findings of this study contribute to a more comprehensive understanding of the early colonization of the gut microbiota.

A total of 17 newborn infants, 11 male, and 6 female were enrolled in this study from May 2023 to June 2023 (Table S1). Among them, 10 were born via eutocia, and 7 were born via C‐section. The average birth weight was 3300.5 ± 476.2 g, with an average gestational age of 38.3 ± 1.5 weeks, and an average maternal age of 32.2 ± 3.5 years. Meconium samples were collected from all infants within 12 h of birth.

The absolute quantification of bacteria was calculated in 34 samples using spike‐in plasmid (Table S5). The average abundance of bacteria in 17 meconium samples and second‐pass feces was found to be 1.14 × 10^7^ and 1.59 × 10^9^ copies/g (CFU/g), respectively (Figure 1A). The top 15 genera present in the meconium were also detected in the second‐pass feces, with 14 out of 15 demonstrating an increase in numbers (Table S2). However, of the 15 genera most abundant in feces, only five were detected in the meconium with a relative abundance greater than 0.05%. The absolute abundance of *Collinsella*, *Serratia*, Erysipelotrichaceae_UCG‐003, *Gemella* in meconium were under 1000 CFU/g. *Serratia* cannot be detected in any of the meconium samples (Table S2).

**Figure 1:**
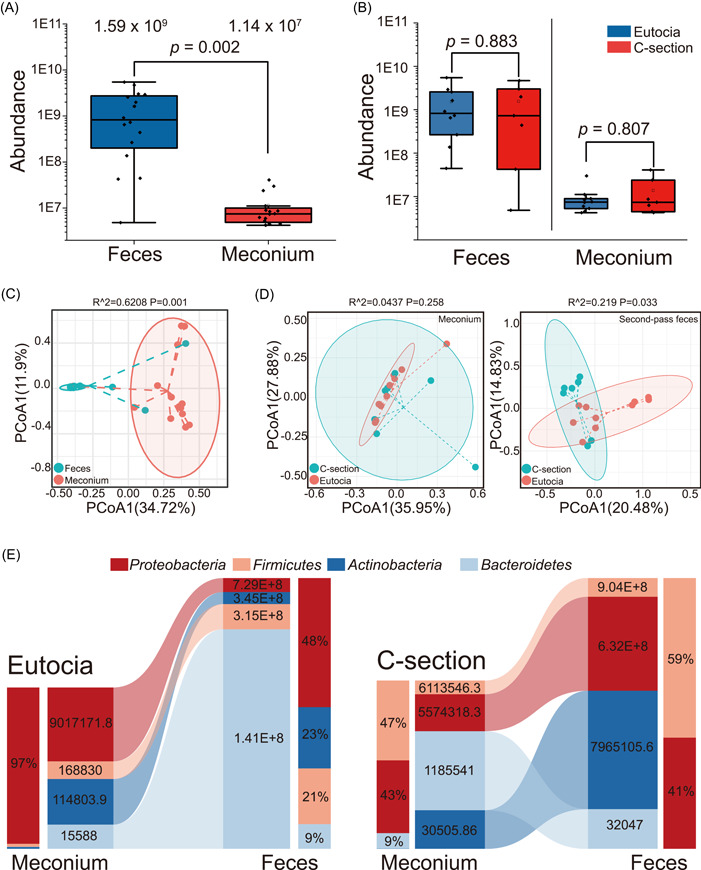
Absolute abundance and PCoA analysis of meconium and second‐pass feces. (A) The absolute abundance of meconium and second‐pass feces (*p* = 0.002). (B) The absolute abundance of eutocia group and C‐section group in meconium and second‐pass feces. There is no significant difference between two groups in meconium and second‐pass feces. (C) PCoA analysis in meconium and feces (*p* = 0.001). (D) PCoA analysis in eutocia group and C‐section group in meconium (left) (*p* = 0.258) and PCoA analysis in eutocia group and C‐section group in second‐pass feces (right) (*p* = 0.033). (E) The absolute abundance and relative abundance changing of Bacteroidetes, Proteobacteria, Actinobacteria, and Firmicutes in the eutocia group (left) and C‐section group (right). PCoA, principal coordinate analysis.

The 34 samples were divided into four groups: eutocia meconium group, C‐section meconium group, eutocia second‐pass feces group, and C‐section second‐pass feces group. The absolute quantification results showed no significant differences in microbial abundance between the eutocia and C‐section groups for both meconium and second‐pass feces (Figure 1B).

According to the beta diversity, The Bray–Curtis distance‐based similarity analysis by principal coordinate analysis indicated that the meconium samples were clustered separately from the second‐pass feces (Figure 1C). Following feeding, the distance of each second‐pass feces sample increased (Figure 1C), indicating that changes in the infant's gut microbiota begin within 72 h of birth.

On the basis of the mode of delivery, both the meconium and second‐pass feces were categorized into eutocia and C‐section groups. The beta diversity analysis revealed no significant difference between the eutocia and C‐section groups in meconium (Figure 1D). However, a significant difference was observed between the eutocia and C‐section groups of second‐pass feces (*p* = 0.033) (Figure 1D).

Considering the mode of delivery, Bacteroidetes, Proteobacteria, Actinobacteria, and Firmicutes emerge as the four most common phyla in the human intestine. At the phylum level, the eutocia group of second‐pass feces mainly consisted of Proteobacteria (47.6%), Actinobacteria (22.6%), Firmicutes (20.6%), and Bacteroidetes (9.2%), while the C‐section group of second‐pass feces primarily comprised Firmicutes (58.2%) and Proteobacteria (40.6%) (Figure 1E).

The number of phyla in the C‐section decreased from the meconium to the second‐pass feces, with reductions observed in certain phyla, such as Bacteroidetes. The Actinobacteria did not take up a big proportion in the C‐section of second‐pass feces, but its average absolute number increased from 30,505.86 CFU/g to 7,965,105.57 CFU/g (Figure 1E).

In this investigation, we employed the spike‐in plasmid method to conduct relative and absolute quantitative analysis of the meconium and the second‐pass feces. The data revealed that the total bacterial count in meconium reached a quantitatively significant level, indicative of the substantial changes in the gut microbiome of newborns within 72 h. Previous studies have primarily focused on longitudinal sampling to examine alterations in infantile gut microbiota [12], with limited quantitative analysis of meconium, a sample of particular significance. Therefore, this study sought to address this gap by providing a quantitative analysis of meconium.

We observed a substantial difference in beta diversity between meconium and second‐pass feces. Each sample in the meconium exhibited a high degree of similarity in beta diversity, which was significantly diminished in the second‐pass feces. Moreover, upon stratifying by mode of delivery, we determined that the similarity of meconium was unaffected by the mode of delivery, whereas a notable distinction was evident between the second‐pass feces of infants delivered via eutocia and those delivered through C‐section.

By employing absolute quantification, we gained a more intuitive understanding of the microbiota composition in meconium and second‐pass feces, as well as the influence of eutocia and C‐section delivery on the infant's gut microbiota. The absolute abundance of microorganisms in second‐pass feces surpassed that of meconium by a fact of 100. Furthermore, while significant interindividual variations were observed in the total microorganism count in second‐pass feces samples, the differences among individual meconium samples were minimal.

When considering the mode of delivery, the result indicates that the mode of delivery did not affect the total number of microbial species, but it did impact the composition of the microbiome. Within 72 h, the gut microbiota of infants born through vaginal delivery (eutocia) more closely resembled that of adults compared to those born by C‐section. In the second‐pass feces, the proportion and number of Actinobacteria and Bacteroidetes in the eutocia group are far more than that of C‐section infants. Additionally, we observed that the changes in the intestinal microbiota were more stable in infants born through eutocia as the abundance of all common phyla increased. In contrast, the changes in the intestinal microbiota of C‐section babies were more disordered, with a decrease in the number of Bacteroidetes, one of the most abundant phyla in the adult intestine.

The presence of microbes in a baby's gut before birth has been a subject of longstanding debate. Kennedy et al. conducted tests on fecal samples of babies delivered by C‐section and reported the absence of bacteria before birth [8]. However, someone may argue that the absence of detection does not necessarily imply non‐existence. The data of our study help to address this concern, because we found an intermediate state (10^6^‐10^7^ CFU/g) between fetuses and adults (10^10^−10^11^ CFU/g) [1]. This discovery addresses a crucial gap in our understanding of the establishment of the human gut microbiota.

## AUTHOR CONTRIBUTIONS

Wen‐Yu Jin, Jing Peng, Jinping Dai, Yi‐Zhou Gao, and Jielin Wang conceived and designed the research. Jing Peng and Jinping Dai provided the samples. Wen‐Yu Jin, Jing Peng, Rongkang Tang, and Jia‐Xin Guo performed experiments. Wen‐Yu Jin, Jinping Dai, and Yi‐Zhou Gao analyzed the data and generated the figures. Wen‐Yu Jin, Jing Peng, and Yi‐Zhou Gao wrote the manuscript. Jinping Dai, Huan Zhao, Jielin Wang, Yi‐Zhou Gao, Rongkang Tang, and Wen‐Yu Jin revised the manuscript. Jing Peng, Jielin Wang, Yi‐Zhou Gao, and Shu Zhang supervised the project. All authors have read the final manuscript and approved it for publication.

## CONFLICT OF INTEREST STATEMENT

The authors declare no conflict of interest.

## ETHICS STATEMENT

The ethics application (No. 2023‐124) was approved by the Research Ethics Committee of the Obstetrics and Gynecology Hospital of Fudan University.

## Supporting information

 


**Table S1**. Type and delivery mode of each sample.
**Table S2**. Top 15 genus in meconium and second‐pass feces.
**Table S3**. The abundance of each spike‐in DNA in different samples.
**Table S4**. Standard curve based on spike‐in DNA.
**Table S5**. The absolute abundance of each sample in genus level.
**Table S6**. The relative abundance of each sample in phylum level.
**Table S7**. The absolute abundance of each sample in phylum level.

## Data Availability

The data that supports the findings of this study are available in the supplementary material of this article. All data analyzed in this study are included here and in its Supporting Information. Supplementary materials (methods, figures, tables, scripts, graphical abstract, slides, videos, Chinese translated version, and update materials) may be found in the online DOI or iMeta Science http://www.imeta.science/.
