# Supplementary material for: Bacterial load in meconium
Source: Imeta. 2024 Feb 13;3(1):e173. doi: 10.1002/imt2.173 (PMC10989067; doi:10.1002/imt2.173)
Supplement: Supplementary file 1 [file IMT2-3-e173-s002.docx]

**Supporting information to Bacterial load in meconium**

**Running title: Bacterial load in meconium**

Wen-Yu Jin^1^#, Jing Peng^2^#, Jinping Dai^2^, Rongkang Tang^1^, Jia-Xin Guo^1^, Huan Zhao^3^, Jielin Wang^4^*, Shu Zhang^5^*, Yi-Zhou Gao^1^*

^1^ The Center for Microbes, Development and Health, Shanghai Institute of Immunity and Infection, Chinese Academy of Sciences, Shanghai, 200031, China

^2^ Obstetrics and Gynecology Hospital of Fudan University, Shanghai, 200090, China

^3^ Department of Oncology, The First Affiliated Hospital of Zhengzhou University, Zhengzhou, 450052, China.

^4^ Hongqiao International Institute of Medicine, Tongren Hospital, Shanghai Jiao Tong University School of Medicine, Shanghai, 200336, China

^5^ Department of Gynecological Oncology, Fudan University Shanghai Cancer Center, Shanghai, 200000, China.

#These authors made same contribution.

*****Corresponding author. Email:

[gaoyizhou@126.com](mailto:gaoyizhou@126.com) (Yi-Zhou Gao); [superdrzhang@yeah.net](mailto:superdrzhang@yeah.net) (Shu Zhang); [wjl4734@shtrhospital.com](mailto:wjl4734@shtrhospital.com) (Jielin Wang)

**SUPPLEMENTARY RESULT**

**Differences in function and composition of the samples**

BugBase is a microbiome analysis tool that identifies high levels of phenotype present in microbiome samples. Based on different sample forms, we found the the bacteria in meconium has less gram-positive form than the second-pass feces (Fig. S1A). For oxygen utilizing, the relative abundance of aerobic bacteria in meconium was higher than in second-pass feces (Fig. S1B). That could be because the newborn infant’s gut is an aerobic environment. The existing study showed that the first step of colonization of bacteria in infants’ gut is the colonization of facultatively aerobic bacteria [1, 2]. This conclusion was also displayed in our result (Fig. S1B). In other phenotypes, the second-pass feces showed higher relative abundance than meconium in mobile elements containing, potentially pathogenetic and stress tolerant (Fig. S1D). The meconium only shows a higher relative abundance in forms biofilms than the second-pass feces (Fig. S1D).

Based on different mode of delivery, the beta diversity of samples from second-pass feces shows a significant different between the eutocia group and C-section group (Fig. 1D). According to the result of BugBase, the eutocia group shows higher in the relative abundance of stress tolerant (*p*=0.029) (Fig. S1C).

**Changes of the gut microbiota in newborns within 72 hours**

From the absolute quantitative results, the 10 bacteria with the largest gap between meconium and second-pass feces were found at the family level. The *Coriobacteriaceae* was the one that has changed the most. The existing study showed that *Coriobacteriaceae* can affect more than 10 metabolisms in adults, but it has seldom been noticed in the infantile gut (Fig. S2A).

*Gemellaceae* was also a huge change between two groups. Only three samples from meconium can be detected in a low level, 12 samples from second-pass feces can be detected with an average of about 2E+06 (Fig. S2A).

*Bifidobacteriaceae* is the most concerned microbia in the infants’ gut. The data shows *Bifidobacteriaceae* became the dominant microbiota in infants’ gut after first fed. It ranked the biggest family in the list except the *Enterobacteriaceae* and *Staphylococcaceae* in second-pass feces. 9 samples were detected *Bifidobacteriaceae* in second-pass feces but only 2 in meconium (Table S3).

**The difference in absolute abundance between eutocia group and C-section group**

In all meconium samples, only one was detected *Bifidobacterium* over the absolute abundance of 1000. In eutocia group of second-passed feces, 6/10 samples were detected. In C-section group, 3/7 samples were detected. The average abundance of *Bifidobacterium* in eutocia group was significantly higher than in C-section group (Fig. S3A). *Escherichia-Shigella* was another genus which showed a significant difference between the eutocia group and C-section group (Fig. S3B).

From the prospective of the whole, the top 10 bacteria in the eutocia group of the second-passed feces were *Escherichia-Shigella*, *Bifidobacterium*, *Veillonella*, *Bacteroides*, *Enterococcus*, *Collinsella*, *Streptococcus*, *Staphylococcus*, *Serratia*, and *Prevotella*. This list was totally different with the C-section group, both in the composition and the abundance (Fig. S3C).

In both two groups, three main genera are *Escherichia-Shigella*, *Bradyrhizobium* and *Burkholderia-Caballeronia-Paraburkholderia*. The absolute abundance of the *Bradyrhizobium* and *Burkholderia-Caballeronia-Paraburkholderia* are almost the same (Fig. S3D). However, through absolute quantification, we can still find some distinction. The *Methanobrevibacter* can be only detected in the C-section group (4/7) (Fig. S3E), this genus of archaea can use H_2_ or formate to reduce CO_2_ to CH_4_ in the gut [3].

**The analysis of alpha diversity**

The alpha diversity can obtain the richness, diversity, and coverage of species in the community. The Sobs, Chao1 and Shannon index both show no significant difference between the meconium and the second-pass feces. (Goods coverage > 0.99) (Fig.S4A & S4B). That means richness and diversity of species in meconium and second-pass feces are almost the same.

From different mode of delivery, the meconium and second-pass feces can also be divided into eutocia group and C-section group. The alpha diversity comparison of these four groups were also done. The sobs index, chao1 index and simpson index of eutocia group and C-section group in meconium and second-pass feces show no significant difference (Fig. S4D-I).

**METHOD**

**Study population and Sampling**

From May 2023 to June 2023, all pregnant women admitted to the obstetrics & gynecology hospital of Fudan University which suitable to the screening conditions were invited to participate in this study. All the pregnant women and newborns which in this study will fit the following conditions, (1) babies were born to healthy mothers; (2) mothers had not received any antibiotic treatment before delivery; (3) mothers had no bowel disease of her own; (4) babies did not require any medical treatment after birth; (5) the first-pass meconium was defecated within 12 hours after birth. Other data including the age of the mothers, the weight of the infants was collected.

The meconium was determined as the first stool after birth and was collected by the nurse in the baby room within the 12 hours after the birth. Fresh meconium samples were obtained from diapers after spontaneous evacuation and stored in a 10ml collecting tube The nurse would collect the fecal sample from the same group of infants within 48 to 72 hours after birth (every infant had been breast-fed). All the samples were transported into refrigerator (-20℃) immediately and stored no more than one week before long term storage at -80 °C.

**DNA extraction and Sequencing**

Total genome DNA from meconium samples was extracted and the concentration was detected by Nanodrop2000 (Thermo Fisher Scientific, Waltham, MA, USA). To ensure that there are target regions of concern in the extracted genomic DNA, the genomic DNA is pre-amplified. The forward primer was 341F: ‘CCTAYGGGRBGCASCAG’, and the reverse primer was 806R: ‘GGACTACNNGGGTATCTAAT’. Then twelve different spike-in DNA with a known copy number diluted according to a specific concentration gradient are added to the sample DNA together as a PCR amplification template. Specific primers with barcode were synthesized and the PCR (Polymerase Chain Reaction) was done with TransStart® FastPfu DNA Polymerase (TransGene Biotech, Beijing, China) using ABI GeneAmp® 9700 (Thermo Fisher Scientific, Waltham, MA, USA). Each sample had 3 copies and extract from the agarose gel by AxyPrep DNA Gel Extraction Kit (Axygen, USA). Then prepare the Novaseq library using TruSeq® DNA Library Prep Kits (Illumina, San Diego, CA, USA). And sequencing was conducted using the Illumina PE300. The sequencing was done by Majorbio (Shanghai, China).

**Absolute quantification**

PE reads obtained by Novaseq sequencing were first spliced according to overlap, and sequence quality was controlled and filtered at the same time. OTU cluster analysis and species taxonomic analysis were performed after samples were distinguished. Based on the OTU clustering results, the spike-in OTU sequence was filtered and extracted, the standard curve of a single sample was constructed, and the absolute copy number of each OTU was calculated according to the fitting formula.

The OTU (Operational Taxonomic Units) was calculated using Uparse (version 11). And then according to the known spike-in DNA sequence information, OTU belonging to spike-in DNA was extracted from the optimized sequence (OTU), and the information of each spike-in DNA in different samples was counted (Table S1). The standard curve was drawn based on the absolute copies of spike-in DNA added to each sample (Table S2) and the abundance value of spike-in DNA obtained by high-throughput sequencing of each sample. The absolute copy number of OTU in each sample can be obtained by inserting the abundance value of OTU in each sample into the standard curve equation:

$$copies number {ng}^{-1}=\frac{OTU total copies number}{template DNA (ng)}$$

*OTU total number: the absolute copy number of OTU obtained by standard curve.*

*Template DNA: the amount of template DNA used in PCR amplification (ng).*

Absolute copy number of OTU in sample:

$$copies number {ng}^{-1}=\frac{OTU total copies number\times extract DNA(ng)}{template DNA \left( ng \right)\times environmental samples (g)}$$

*OTU total number: the absolute copy number of OTU obtained by standard curve.*

*template DNA: the amount of template DNA used in PCR amplification (ng).*

*extract DNA:* *the total amount of DNA extracted (ng).*

*environmental sample:* *the amount of samples used during DNA extraction (g)*

The OTU corresponding species classification information was analyzed using QIIME [4] and RDP Classifier (version 2.2) [5]. Consider that each microbe has more than one copy of 16S rDNA, the gene copy number of each OTU was estimated based on the level of closest relative species based on the rrnDB database (V5.8) [6], and then the absolute copy number of the OTU was divided by the gene copy number to calculate the CFU in the samples.

**Data Analysis**

The Alpha diversity was analysis using the R software. The parameter of analysis including Sobs and Shannon index[7]. The Wilcoxon rank sum test was used to test differences between groups. The Bar plot of different levels was analysis using the R software including the relative abundance of each species in the sample and the absolute abundance of each species in the sample. The Wilcoxon rank sum test was used to detect differences between groups.

Principal coordinates analysis (PCoA) [8] and non-metric multidimensional scaling (NMDS) [9] were based on the Bray–Curtis distance matrix.

BugBase is a microbiome analysis tool that identifies high levels of phenotype present in microbiome samples, enabling phenotypic prediction [10]. BugBase first normalizes the OTU with the predicted 16S copy number and then predicts the microbial phenotype using the pre-calculated file provided. Phenotypes include Gram Positive, Gram Negative, Biofilm Forming, Pathogenic, Mobile Element Containing, oxygenating requirements (including aerobic, anaerobic, and facultatively anaerobic) and oxidative stress tolerance.

**
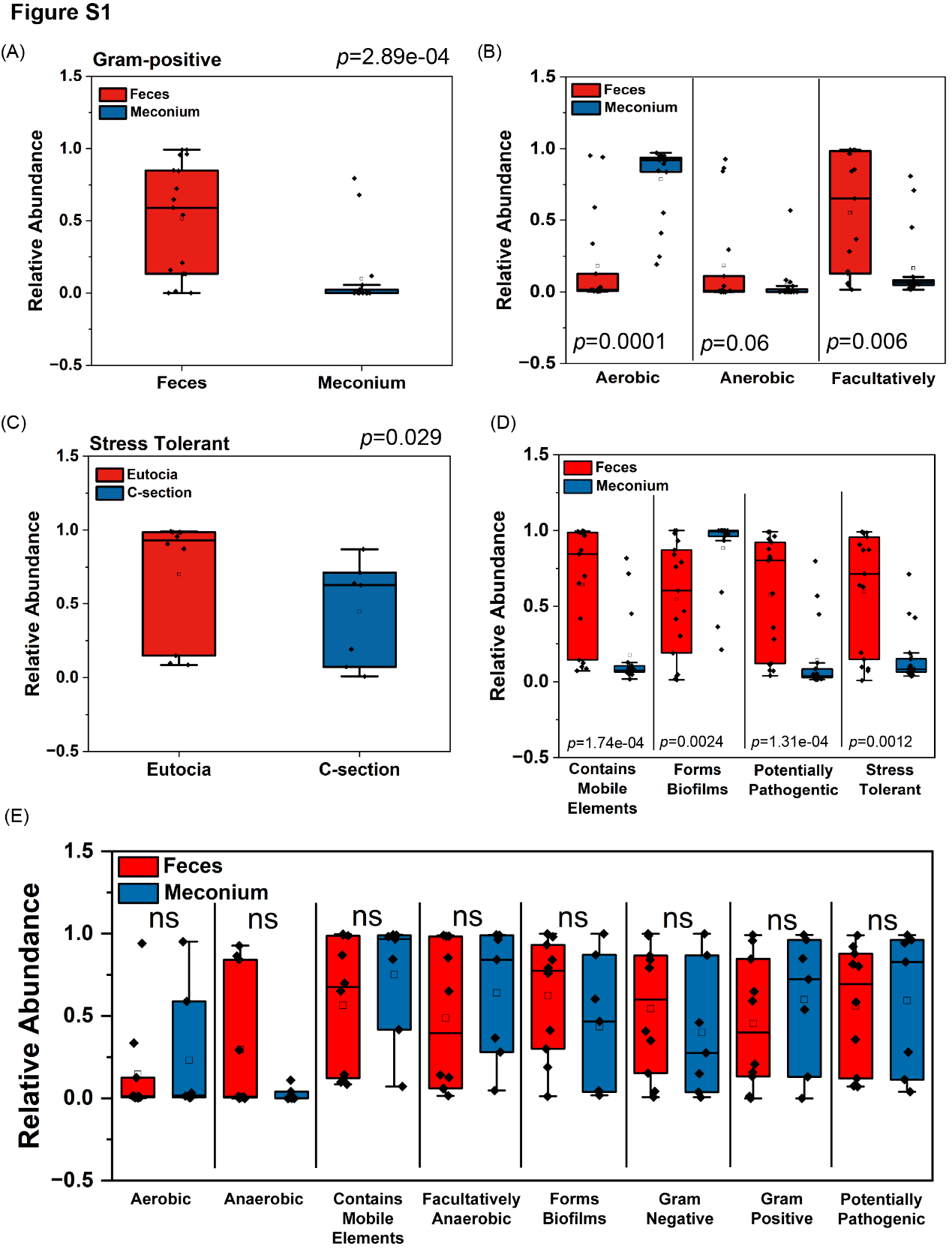
Figure S1** **BugBase analysis based on the sequencing dataset** (A-B): Prediction of phenotypic differences from 16S rDNA sequence data associated with aerobic and Gram-positive bacteria from meconium and second-pass feces. (C): Prediction of phenotypic differences from 16S rDNA sequence data associated with stress tolerance from eutocia group and C-section group in second-pass feces. (D): Prediction of phenotypic differences from 16S rDNA sequence data associated with potentially pathogenic, stress tolerance, mobile element and biofilms formation from meconium and second-pass feces. (E): Prediction of phenotypic differences from 16S rDNA sequence data associated with aerobic, potentially pathogenic, mobile element, biofilms formation, Gram-negative bacteria, and Gram-positive bacteria from eutocia group and C-section group in second-pass feces.

**Figure S2** The absolute abundance results of the 10 bacteria with the largest gap between meconium and second-pass feces in family level. **
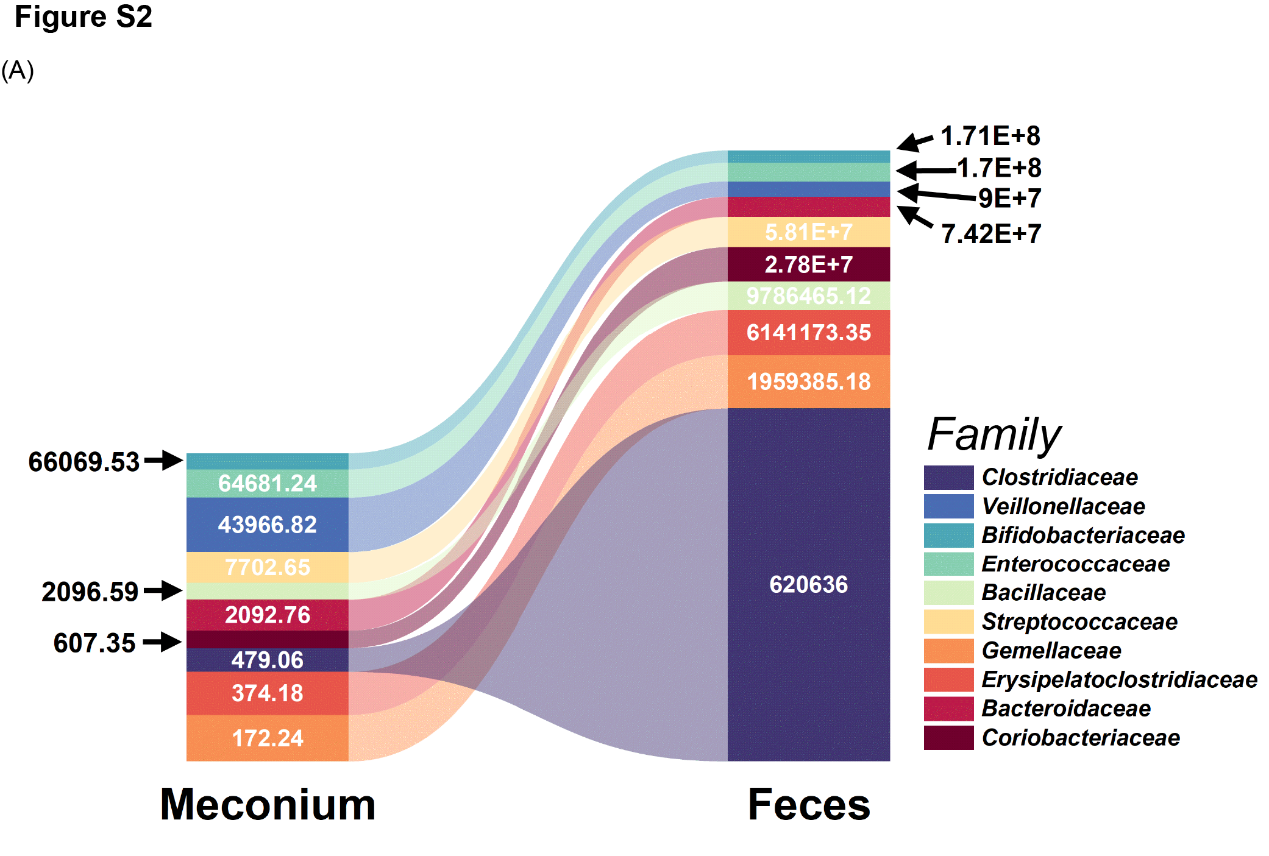
**

**
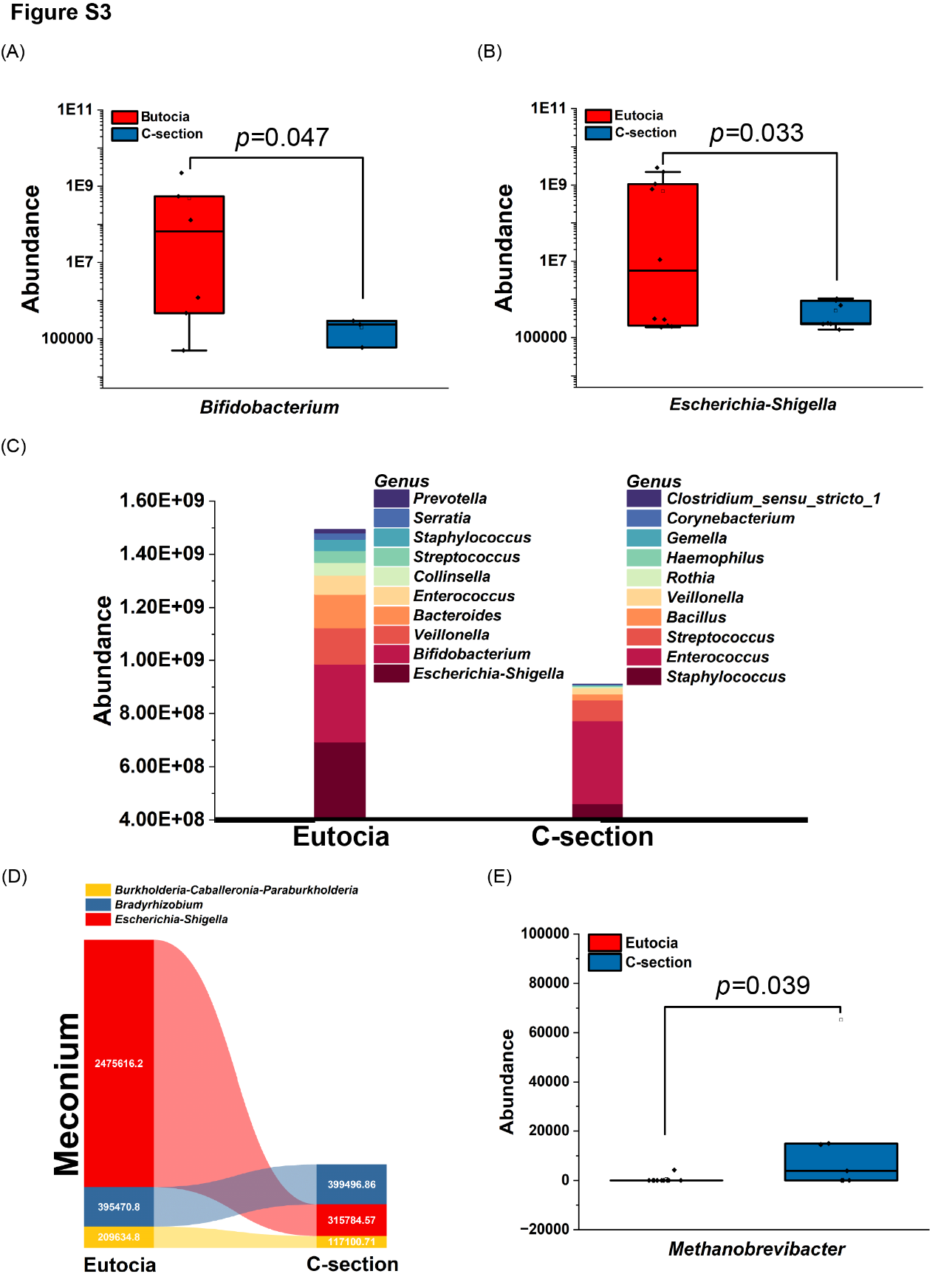
Figure S3** **The difference in absolute abundance between eutocia group and C-section group (**A) The *Bifidobacterium* of eutocia group and C-section group in second-pass feces. (*p*=0.047) (B) The *Eschericha-Shigella* of eutocia group and C-section group in second-pass feces. (*p*=0.033) (C) The absolute abundance of top 10 genus in eutocia group and C-section group of second-pass feces. (D) The absolute abundance of *Bradyrhizobium*, *Burkholderia-Caballeronia-Paraburkholderia* and *Escherichia-Shigella* changes from the eutocia group to C-section group of meconium. E: The absolute abundance of *Methanobrevibacter* in eutocia group and C-section group of meconium.

**
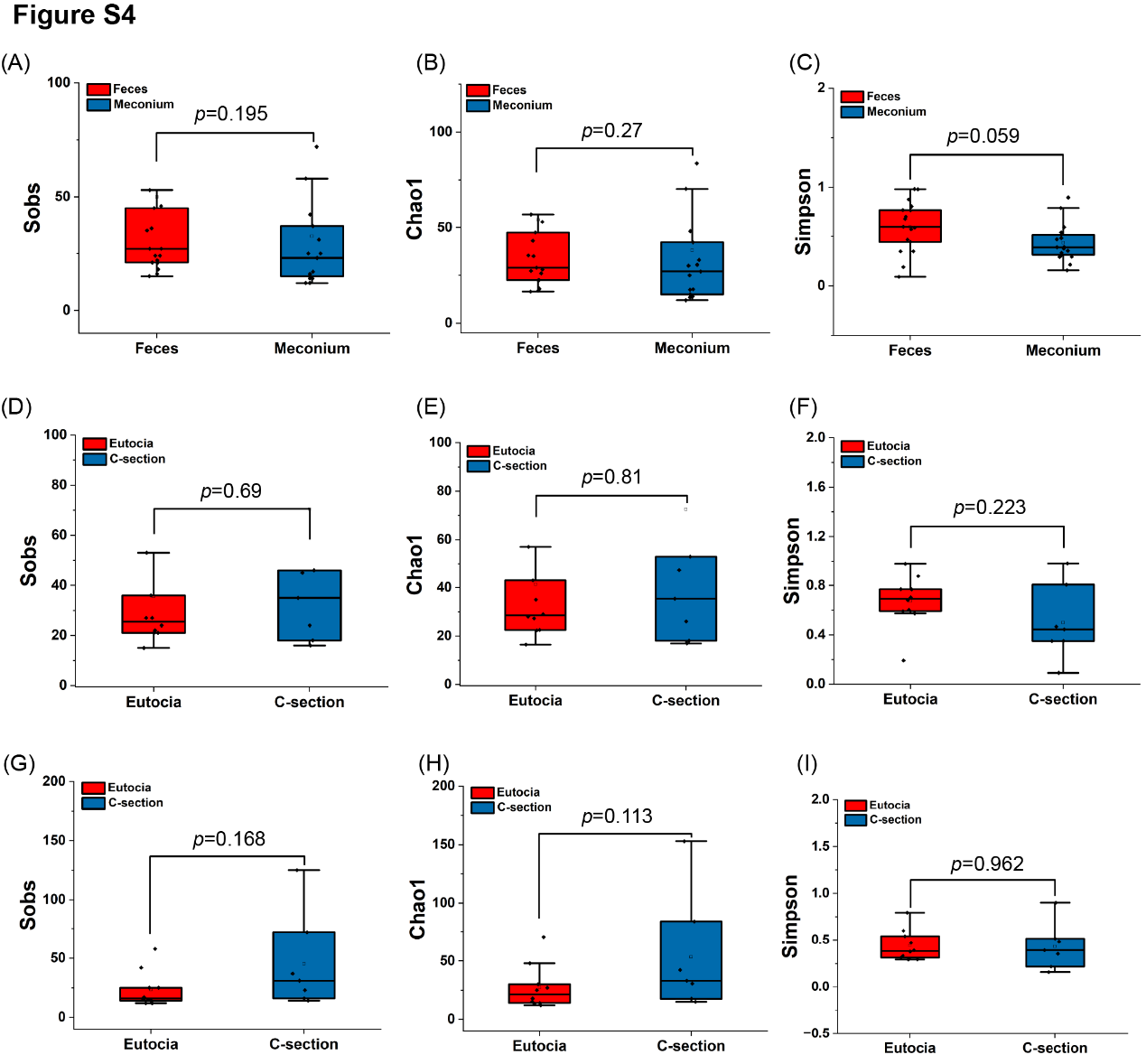
Figure S****4** **The analysis of alpha diversity** (A) The sobs index of second-pass feces and meconium. (*p*=0.195) (B) The chao1 index of second-pass feces and meconium. (*p*=0.27) (C) The Simpson index of second-pass feces and meconium. (*p*=0.059) (D) The sobs index of eutocia group and C-section group in meconium. (*p*=0.69) (E) The chao1 index of eutocia group and C-section group in meconium. (*p*=0.81) (F) The simpson index of eutocia group and C-section group in meconium. (*p*=0.223) (G) The sobs index of eutocia group and C-section group in second-pass feces. (*p*=0.168) (H) The chao1 index of eutocia group and C-section group in second-pass feces. (*p*=0.113) (I) The simpson index of eutocia group and C-section group in second-pass feces. (*p*=0.962)

**REFERENCE**

1. Palmer, Chana, Elisabeth M Bik, Daniel B DiGiulio, David A Relman, Patrick O Brown. 2007. “Development of the human infant intestinal microbiota.” *PLoS Biol* 5: e177. <https://doi.org/10.1371/journal.pbio.0050177>

2. Mulligan, Christopher M, Jacob E Friedman. 2017. “Maternal modifiers of the infant gut microbiota: metabolic consequences.” *J Endocrinol* 235: R1-r12. <https://doi.org/10.1530/joe-17-0303>

3. Mafra, Denise, Marcia Ribeiro, Larissa Fonseca, Bruna Regis, Ludmila F M F Cardozo, Henrique Fragoso Dos Santos, Hugo Emiliano de Jesus, et al. 2022. “Archaea from the gut microbiota of humans: Could be linked to chronic diseases?” *Anaerobe* 77: 102629. <https://doi.org/10.1016/j.anaerobe.2022.102629>

4. Bolyen, Evan, Jai Ram Rideout, Matthew R. Dillon, Nicholas A. Bokulich, Christian C. Abnet, Gabriel A. Al-Ghalith, Harriet Alexander, et al. 2019. “Reproducible, interactive, scalable and extensible microbiome data science using QIIME 2.” *Nature Biotechnology* 37: 852-857. <https://doi.org/10.1038/s41587-019-0209-9>

5. Cole, James R., Qiong Wang, Jordan A. Fish, Benli Chai, Donna M. McGarrell, Yanni Sun, C. Titus Brown, Andrea Porras-Alfaro, Cheryl R. Kuske, James M. Tiedje. 2013. “Ribosomal Database Project: data and tools for high throughput rRNA analysis.” *Nucleic Acids Research* 42: D633-D642. <https://doi.org/10.1093/nar/gkt1244>

6. Quast, Christian, Elmar Pruesse, Pelin Yilmaz, Jan Gerken, Timmy Schweer, Pablo Yarza, Jörg Peplies, Frank Oliver Glöckner. 2013. “The SILVA ribosomal RNA gene database project: improved data processing and web-based tools.” *Nucleic Acids Res* 41: D590-596. <https://doi.org/10.1093/nar/gks1219>

7. Schloss, Patrick D, Sarah L Westcott, Thomas Ryabin, Justine R Hall, Martin Hartmann, Emily B Hollister, Ryan A Lesniewski, et al. 2009. “Introducing mothur: open-source, platform-independent, community-supported software for describing and comparing microbial communities.” *Appl Environ Microbiol* 75: 7537-7541. <https://doi.org/10.1128/aem.01541-09>

8. Tap, Julien, Muriel Derrien, Hans Törnblom, Rémi Brazeilles, Stéphanie Cools-Portier, Joël Doré, Stine Störsrud, Boris Le Nevé, Lena Öhman, Magnus Simrén. 2017. “Identification of an Intestinal Microbiota Signature Associated With Severity of Irritable Bowel Syndrome.” *Gastroenterology* 152: 111-123.e118. <https://doi.org/10.1053/j.gastro.2016.09.049>

9. Dombrowski, Nina, Klaus Schlaeppi, Matthew T Agler, Stéphane Hacquard, Eric Kemen, Ruben Garrido-Oter, Jörg Wunder, George Coupland, Paul Schulze-Lefert. 2017. “Root microbiota dynamics of perennial Arabis alpina are dependent on soil residence time but independent of flowering time.” *Isme j* 11: 43-55. <https://doi.org/10.1038/ismej.2016.109>

10. Thomas, Andrew M, Eliane C Jesus, Ademar Lopes, Samuel Aguiar Jr, Maria D Begnami, Rafael M Rocha, Paola Avelar Carpinetti, et al. 2016. “Tissue-Associated Bacterial Alterations in Rectal Carcinoma Patients Revealed by 16S rRNA Community Profiling.” *Front Cell Infect Microbiol* 6: 179. <https://doi.org/10.3389/fcimb.2016.00179>
